# Supplementary figures and images for: The multifaceted role of the viral 2A protease in enterovirus replication and antagonism of host antiviral responses
Source: PLoS Pathog. 2025 Aug 28;21(8):e1013443. doi: 10.1371/journal.ppat.1013443 (PMC12410887; doi:10.1371/journal.ppat.1013443)

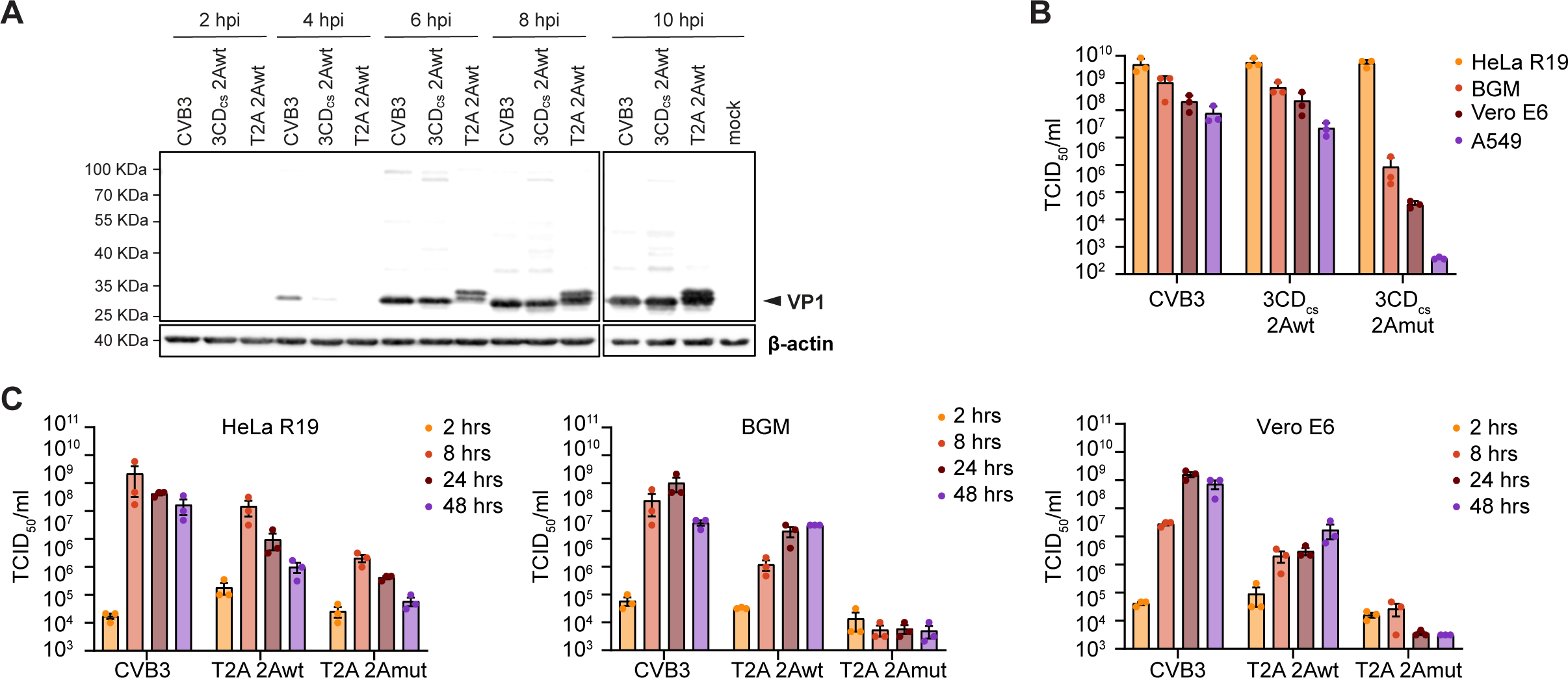

Supplement: S1 Fig — (A) Assessment of viral polyprotein processing efficiency by western blot analysis of CVB3 VP1 protein and β-actin. HeLa-R19 cells, (mock-)infected with CVB3, 3 CDcs-2Awt, T2A-2Awt at an MOI of 10, were lysed at 2, 4, 6, 8 and 10 hpi. (B) CVB3, 3 CDcs-2Awt, and 3 CDcs-2Amut viral stocks were titrated on HeLa-R19, BGM, Vero E6 and A549 by end-point dilution. (C) Growth curves of CVB3, T2A-2Awt and T2A-2Amut viruses in HeLa-R19, BGM, Vero E6 and A549 cells performed as in Fig 1E. Data represent the mean ± SEM of the three technical replicates (B and C). Related to Fig 1. (TIF) [file ppat.1013443.s002.tif]

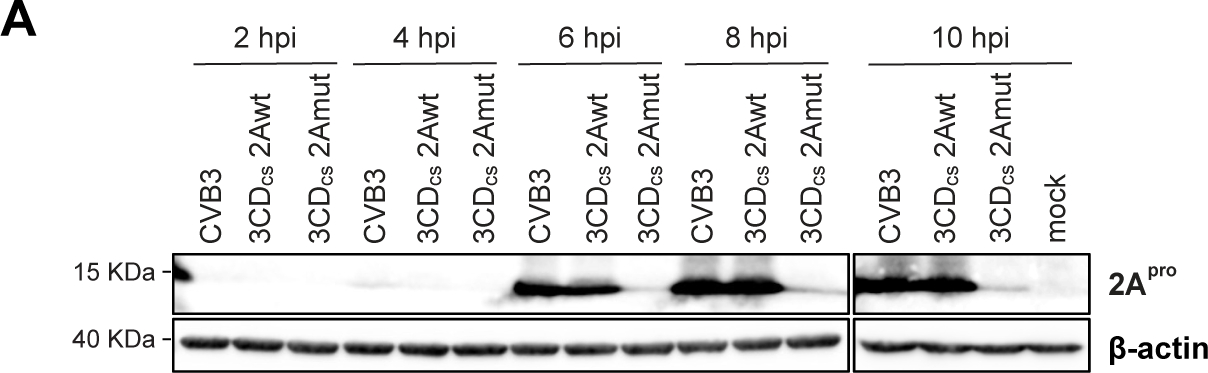

Supplement: S2 Fig — Western blot analysis for CVB3 2Apro. HeLa-R19 cells, (mock-)infected with CVB3, 3 CDcs-2Awt, or 3 CDccs-2Amut at an MOI of 10, were lysed at 2, 4, 6, 8 and 10 hpi. Related to Fig 3A. (TIF) [file ppat.1013443.s003.tif]

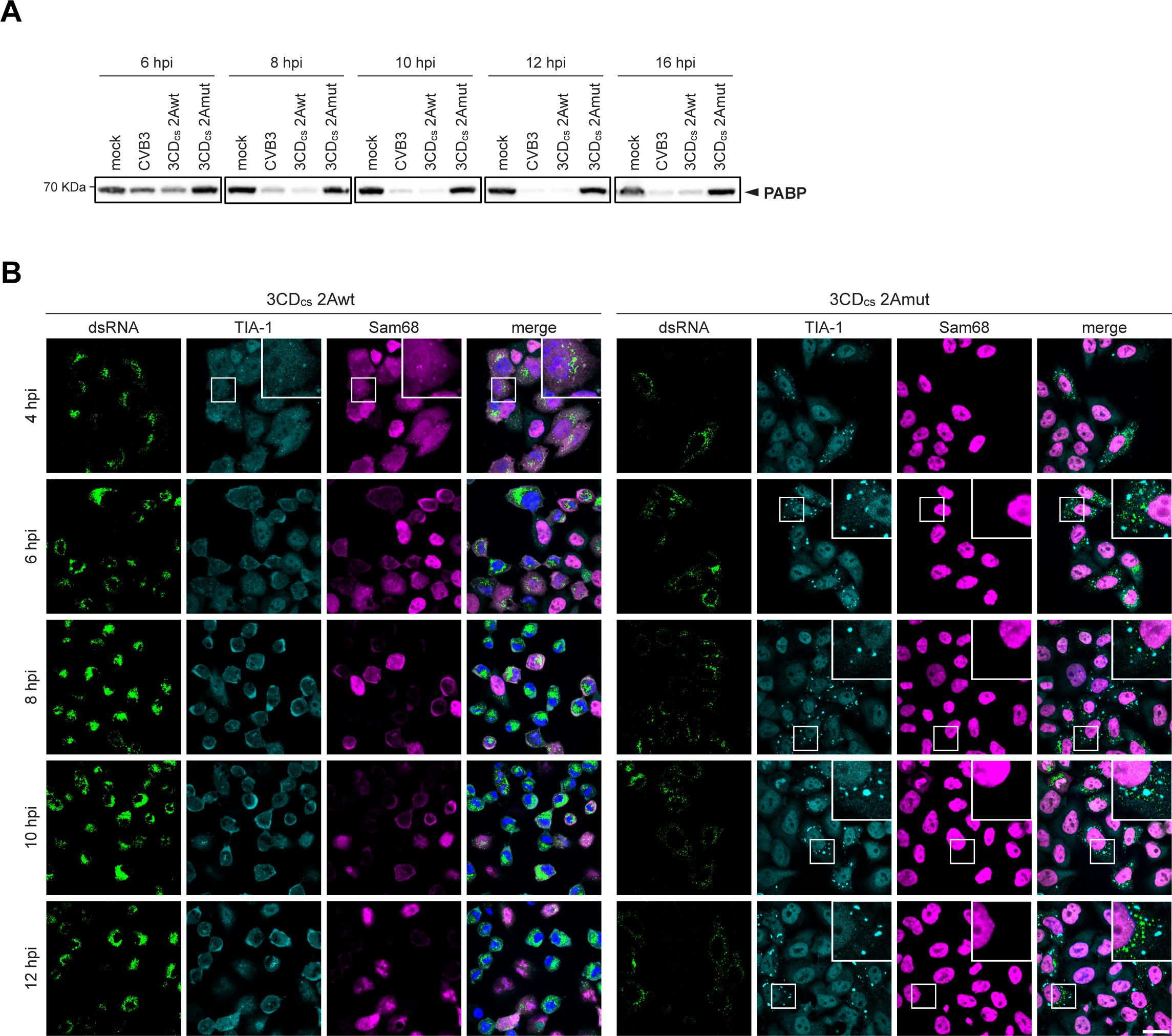

Supplement: S3 Fig — (A) Western blot analysis for PABP. Cell lysates derive from the same experiment shown in Fig 4D. (B) IF analysis of HeLa-R19 cells infected with CVB3, 3 CDcs-2Awt and 3 CDcs-2Amut viruses, fixed and stained for dsRNA as infection marker and TIA-1 and Sam68 as SG marker. Experiment was performed as described in Fig 4C. Magnified regions are shown in white boxes. Scale bar: 25 μm. Related to Fig 4. (TIF) [file ppat.1013443.s004.tif]

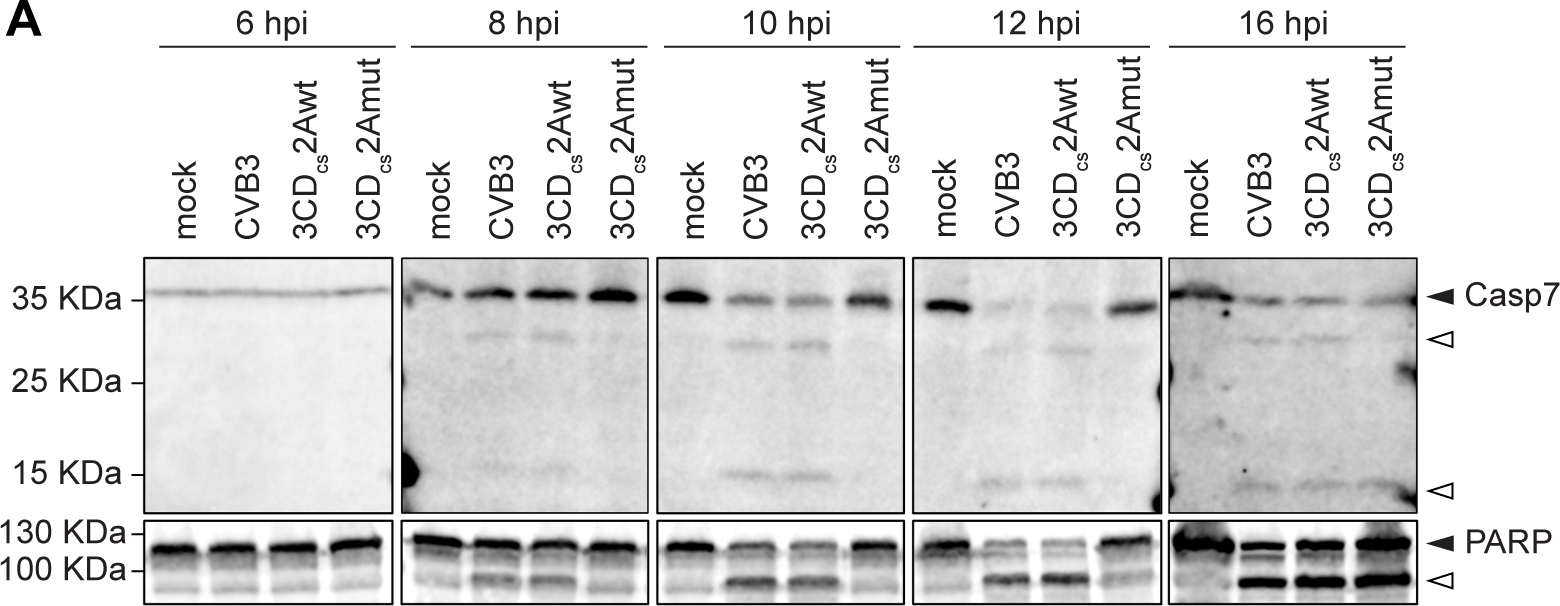

Supplement: S4 Fig — (A) Western blot analysis of caspase 7 (Casp7) and PARP using cell lysates corresponsing to the same experiment shown in Fig 4D. Black triangles mark intact proteins, white triangles the corresponding cleavage products. Related to Fig 5. (TIF) [file ppat.1013443.s005.tif]

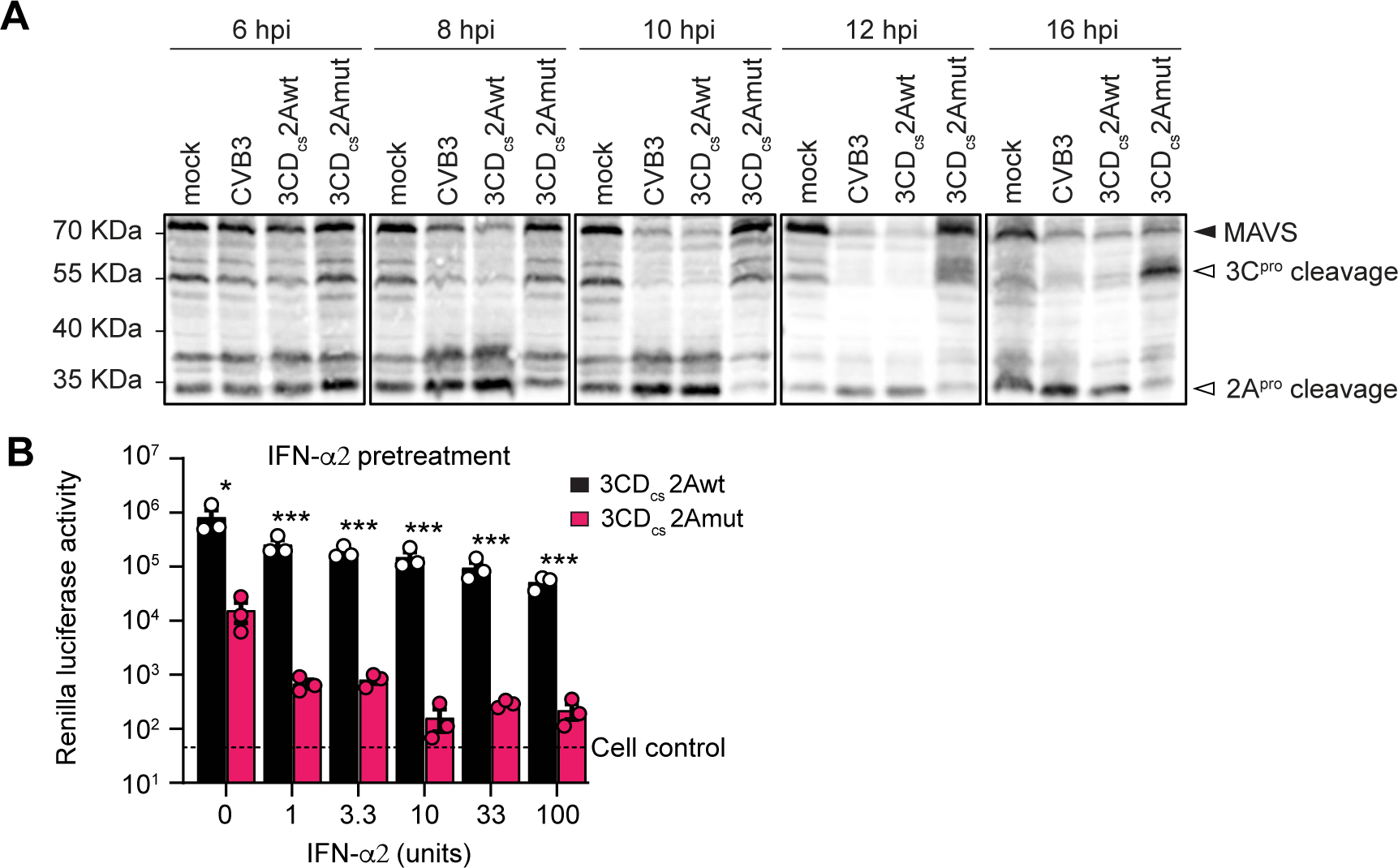

Supplement: S5 Fig — (A) Western-blot analysis of MAVS in cell lysates obtained from the same experiment shown in Fig 4D. Black triangles denote intact proteins, white triangles the cleavage products. (B) non-normalized dataset as represented in Fig 6C. Statistical significance was assessed by multiple two-tailed unpaired t-tests with multiple comparisons corrections using the Bonferroni-Dunn method (* p < 0.05; ** p < 0.01; *** p < 0.001). Related to Fig 6. (TIF) [file ppat.1013443.s006.tif]

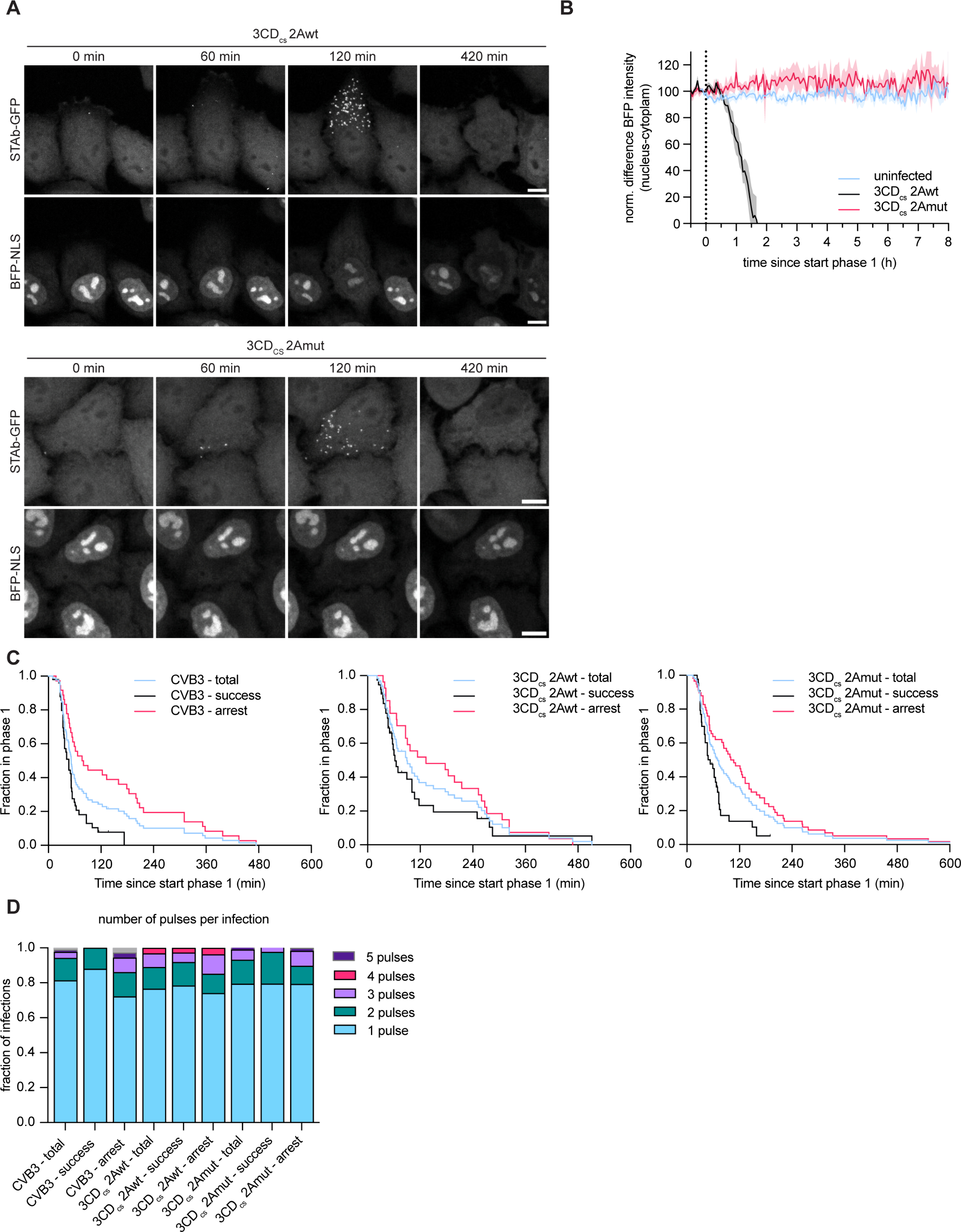

Supplement: S6 Fig — Hela-R19 saGFP-STAb BFP-NLS C1 cells were infected at an MOI of 0.25 with ST-CVB3–2Awt or ST-CVB3–2Amut and time-lapse imaging of STAb-GFP and BFP-NLS was performed. (A) Representative pictures of cells infected with ST-CVB3–2Awt or -2Amut at different time points aligned to the start of phase 1. (B) average normalized BFP-NLS intensity ratio between nucleus and cytoplasm over time aligned to start phase 1. Scale bar: 10 μm. (C) Kaplan-Meier curves depicting duration of phase 1 in those cells with successful replication, non-successful replication (i.e., phase 2 arrested), or both (total). (D) Fraction of infections with indicated number of translation pulses. Related to Fig 7. (TIF) [file ppat.1013443.s007.tif]

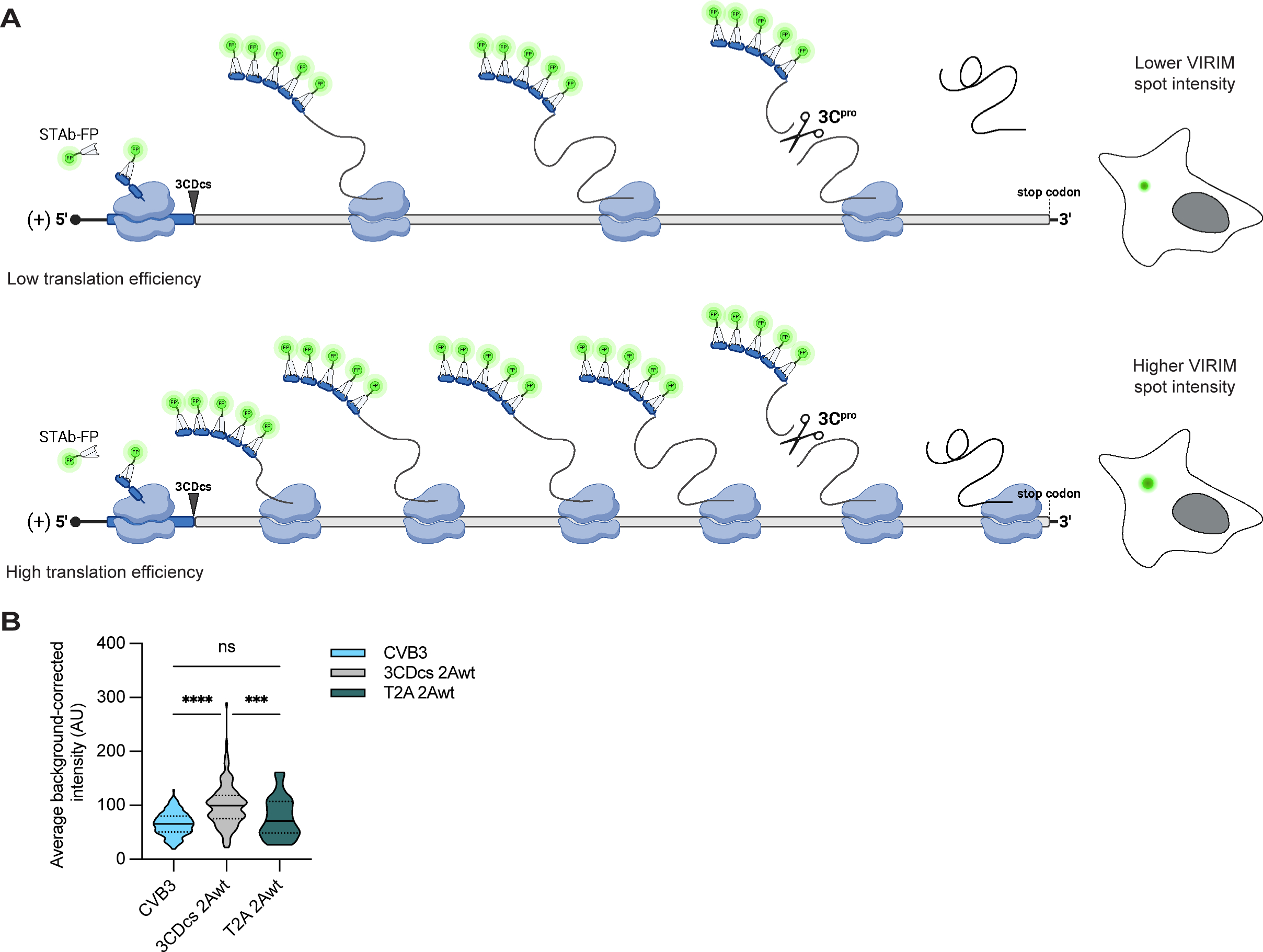

Supplement: S7 Fig — (A) Schematic representation of VIRIM spot intensity analysis. Upper panel shows a situation with comparatively lower translation efficiency, less translating ribosomes on a single vRNA, and consequently a smaller accumulation of fluorescence signal. The lower panel depicts the scenario of a comparatively higher translation efficiency. (B) Absolute intensity measurements of different CVB3 mutants. The higher intensity for 3 CDcs-2Awt is the consequence of a relatively longer effective transcript length (i.e., the length of transcript that is decoded before the nascent peptide chain is released from the ribosome). statistical significance was assessed by a one-way analysis of variance (ANOVA) with multiple comparisons testing. * p < 0.5; ** p < 0.01; *** p < 0.001. (A) created in BioRender. Schipper, J. (2025) https://BioRender.com/unbzo59. Related to Fig 8. (TIF) [file ppat.1013443.s008.tif]

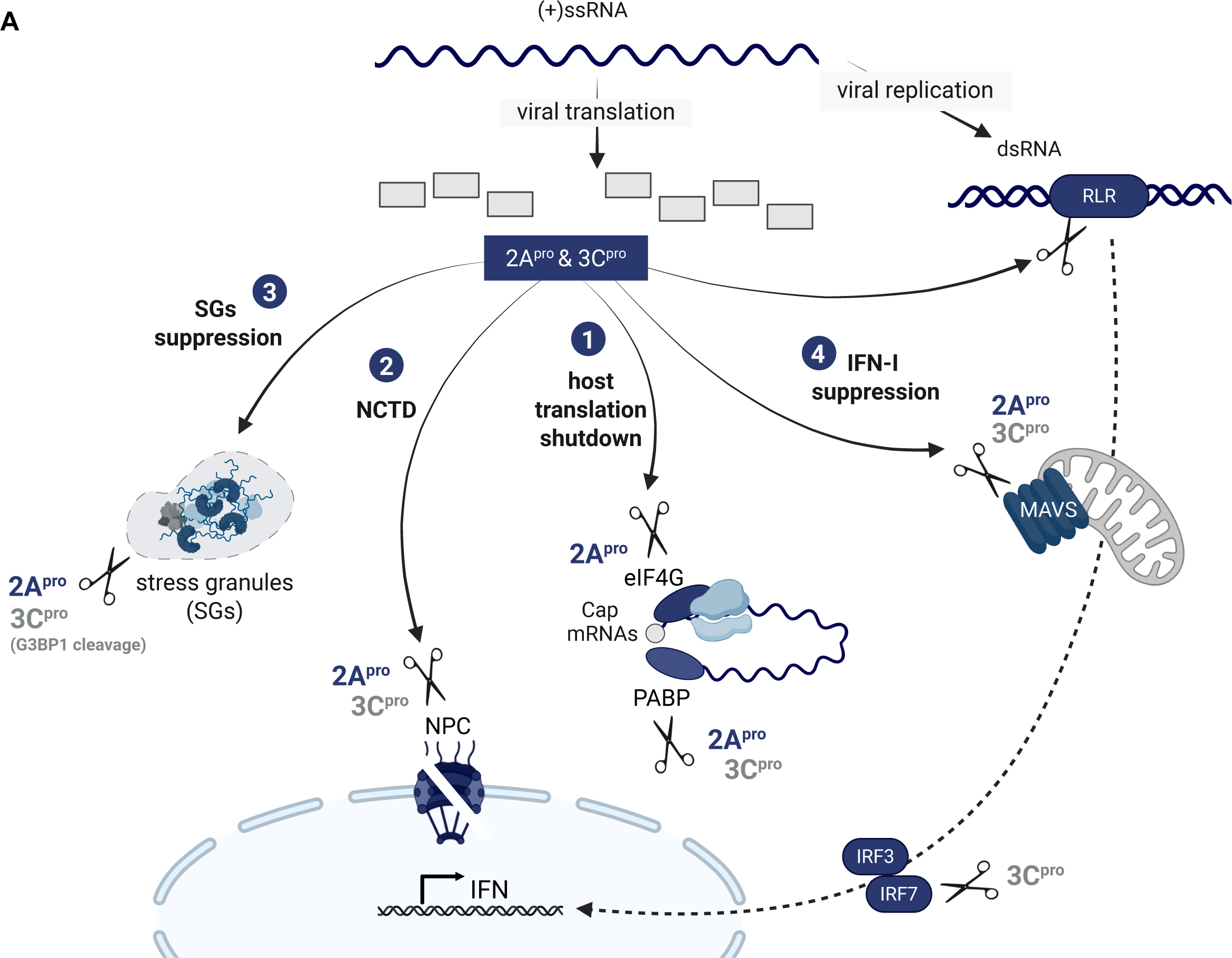

Supplement: S8 Fig — Schematic depiction of the different functions of 2Apro and 3Cpro. (1) 2Apro is critical for establishing the host translation shutdown observed in enterovirus-infected cells, via cleavage of eIF4G and PABP. Even though 3Cpro was previously also found to cleave PABP, we were not able to detect this cleavage during CVB3–2Amut infection. (2) 2Apro, and not 3Cpro, is essential for the rapid establishment of a nucleocytoplasmic trafficking disorder (NCTD), via the cleavage of nucleoporins. We can however not fully exclude that 3Cpro might also subtly contribute to the induction of the NCTD. (3) 2Apro catalytic activity is required for preventing the formation of stress granules (SGs) during enterovirus infection, although the mechanism remains unclear. G3BP1 cleavage by 3Cpro does not seem to be required for SG inhibition. (4) 2Apro cleaves MAVS and MDA5. Interestingly, we were able to detect 3Cpro-mediated cleavage of MAVS, which was previously reported, only at much later timepoints. Moreover, the temporal dynamics of MAVS and MDA5 cleavage raise questions about their relevance in preventing the induction of IFN signaling. We hypothesize that the 2Apro-mediated NCTD might play a previously under-appreciated role in suppressing IFN signaling. Even though from our study it appears that 3Cpro is less important than 2Apro for the depicted effects, we cannot exclude that 3Cpro might still contribute. Created in BioRender. Schipper, J. (2025) https://BioRender.com/fexc61o. (TIF) [file ppat.1013443.s009.tif]
