# Supplementary material for: The multifaceted role of the viral 2A protease in enterovirus replication and antagonism of host antiviral responses
Source: PLoS Pathog. 2025 Aug 28;21(8):e1013443. doi: 10.1371/journal.ppat.1013443 (PMC12410887; doi:10.1371/journal.ppat.1013443)
Supplement: Attachment — Submitted filename: Revised_2A-manuscript_Response to reviewers.pdf [file ppat.1013443.s010.pdf]

Universiteit Utrecht

Faculty of Veterinary Medicine

Prof. Dr. Frank van Kuppeveld

Head Section of Virology,
Division of Infectious Diseases & Immunology,
Department of Biomolecular Health Sciences,
Faculty of Veterinary Medicine,
Utrecht University

Androclus building, Room 512, Yalelaan 1, 3584 CL Utrecht, The Netherlands

Phone +31 30 253 4173, Fax +31 30 253 6723,
E-mail: f.j.m.vankuppeveld@uu.nl

Dear editor and reviewers,

First of all, thank you for the careful assessment of our manuscript and for coming up with possible improvements and questions for clarification. We have tried to address all the points raised, and detail our response point-by-point below. We hope that with the additional data and modifications that we have included our manuscript will meet the standards for publication in PLOS Pathogens.

Yours sincerely,
Prof. Frank van Kuppeveld

EDITORIAL

1. Check whether figure 2A is original or whether it was made with BioRender. If so, refer to BioRender.
   We have addressed all the Biorender citations, and have sent publication licenses (via email) to the editor, as well as included them in the raw data file.
2. Financial disclosure form needs to be looked at:
   Please amend your detailed Financial Disclosure statement. This is published with the article. It must therefore be completed in full sentences and contain the exact wording you wish to be published. Please ensure that the funders and grant numbers match between the Financial Disclosure field and the Funding Information tab in your submission form. Note that the funders must be provided in the same order in both places as well. State what role the funders took in the study. If the funders had no role in your study, please state: "The funders had no role in study design, data collection and analysis, decision to publish, or preparation of the manuscript.". If you did not receive any funding for this study, please simply state: u201c The authors received no specific funding for this work. u201d
   We have addressed this.

REVIEWER COMMENTS

Reviewer 1 (Honglin Luo)

<u>Points from Summary</u>

This study investigates the role of the enteroviral 2A protease in viral replication and immune evasion, using engineered coxsackievirus B3 (CVB3) with inactive 2A via alternative cleavage sequence on viral genome. The authors demonstrate that 2A is critical for efficient replication in vitro and in vivo, and for disrupting host defenses including nucleocytoplasmic transport, mRNA translation, stress granule formation, and interferon responses. Using single-molecule live-cell imaging, they also show that 2A is required for the early replication of incoming viral RNA. The study cleverly addresses a major experimental challenge by enabling the functional analysis of the 2A protease in the context of infection. The approach is innovative and yields valuable insights into the multifaceted role of 2A in viral replication and host interaction. However, several points require clarification or additional data to strengthen the conclusions:

Major comments

1. Statistical analyses are missing in several figures, such as Figures 1, 2C and 6C. Please provide appropriate statistical evaluations to support the conclusions.
   We acknowledge that these were missing and have now included additional statistical analyses. For figure 1, we noticed that we previously had made a small error in the method for normalization (we previously normalized to individual replicates at t=0, whereas now we normalize to the average of the three replicates at t=0) which has now been corrected. The raw data includes excel files used for normalization and for statistical analyses.

2. Quantification of Western blot data is lacking. Please include densitometric analysis for all Western blots presented in Figures 3A, 4A–B, 4D, and 6B to allow for proper assessment of protein expression and cleavage efficiency. For instance, in lines 347-349, the statement "Interestingly, we observed earlier and more pronounced PKR and eIF2α phosphorylation in CVB3-2Amut- than in CVB3-2Awt-infected cells (Figure 4A) …" is not fully supported by the results in the absence of data quantification.
   We have added densitometric analyses of the western blots in the paper in supplemental data table 1 to support the conclusions drawn. Also, we have altered our statement on PKR and eIF2alpha phosphorylation accordingly.

3. In Figure 1B, can the authors detect the P1 and P2 cleavage fragments directly by Western blot to assess the efficiency of polyprotein processing among the three recombinant CVB3 viruses containing WT-2A?
   We have performed a WB analysis using an anti-VP1 antibody to detect polyprotein processing of the P1-P2 region, and included this in supplemental figure 1. Overall, polyprotein processing appeared to be efficient, as there is no accumulation of intermediate products. According to expectations, we observed a somewhat larger band for CVB3-T2A-2Awt, in line with the additional length of the T2A sequence. The reason for the observation of double VP1 bands in the CVB3-T2A-2Awt condition is unknown.

   The observation that viral RNA levels remain unchanged while viral titers are greatly reduced between WT-CVB3 and mutant-CVB3 (Figure 1B), in contrast to the opposite trend seen in Figure 2B, needs further clarification beyond the possibility of inefficient polyprotein cleavage.
   We acknowledge the reviewer's point, but would like to emphasize that the comparison of WT-CVB3 and mutant-CVB3 in Fig. 2B concerns WT-CVB3 with 3CDcs-2Awt in the *in vivo* setting. Looking at that same comparison in Fig. 1B (i.e., in HeLa cells), we see a slight reduction in infectious titers, and an even smaller reduction in vRNA (whereas this is a much larger difference for the comparison between WT-CVB3 and T2A-2Awt, for which we suggested it might relate to subtle differences in polyprotein cleavage). As for why the effects on vRNA and viral titers might be larger in the *in vivo* setting, this might reflect the more complex context, where effects on replication efficiency of the virus might be more pronounced. This is supported by the observation of large differences between different cell types (e.g., A549 vs HeLa in Fig. 1E).

   Moreover, could the authors comment on the typical duration of a single replication cycle for these viruses in HeLa cells? Is it completed within 16 hours?
   A single replication cycle of wild-type CVB3 in HeLa cells takes about 8-10 hours, and is thus completed within 16 hours. This is supported by the plateauing of the vRNA levels and infection titers by 10 hpi, as observed in Fig. 1B. We have indicated this in the text now: *"A full infection cycle of CVB3 on HeLa cells takes roughly 8-10 hours."*

4. In Figure 1E, the authors should discuss why the 3CD-2Amut virus produced much lower viral titers in BGM, Vero E6, and A549 cells compared to 3CDcs-2Awt, while this difference was less pronounced in HeLa R19 cells. What factors might account for the cell-type dependent differences in viral replication or susceptibility?
   We agree with the reviewer that this is an important point of discussion, and have referred to it in the discussion and limitations sections. However, we have tried to emphasize it more already in the main text by adding the following:
   "The reason for the divergent importance of 2Apro catalytic activity in different cell lines remains unclear, but it might relate to steady-state expression levels of specific intrinsic restriction factors that are countered by 2Apro. Alternatively, 2Apro activity might be more or less important to facilitate a pro-viral host environment in different cell lines."

5. Figure 4A, data for PKR cleavage is not convincing. The authors should show cleavage of PKR total protein which looks unchanged across the different infections.
   Although cleavage of PKR by $3C^{pro}$ has been reported in the past (Black et al., 1989), and we were able to detect cleavage fragments, we were not able to detect differences in total PKR protein levels, and thus likely only reflects a small pool of the total PKR being cleaved. We have adapted our text to align more with this:
   "We could detect cleavage fragments of PKR during both 3CDcs-2Awt and 3CDcs-2Amut infection (Fig 4A), reflecting cleavage of PKR by $3C^{pro}$, a well-known phenomenon observed in enterovirus-infected cells [43]. However, we were not able to detect any clear differences in the pool of full-length PKR, implying that only a small subset is cleaved. Moreover, this cleavage occurred only late in infection, after PKR activation and eIF2α phosphorylation, and thereby likely has little, if any, effect on ISR activation."

6. In Figure 4B, viral protein translation is clearly observed in the 3CDcs-2Awt group at 5–6 hours post-infection. However, by 7 hours and beyond, the viral protein bands appear to diminish or disappear. Could the authors clarify the reason for this decline? This point is also relevant to Question 3 regarding the duration of the viral life cycle in HeLa cells.
   The experiment represented in Fig. 4B queries active translation, and it is generally accepted that towards the end of the viral infection cycle the virus shifts away from translation. The mechanism behind this is not fully understood, and it is not known whether this relates to a switch from translation and replication towards assembly, or whether it is the consequence of the initiation of death programs on cellular translation machinery. We have added the following sentence:
   "At later timepoints during infection, viral IRES-dependent translation decreases, as is reflected by the decrease in active translation we observed from 7hpi onwards. It is not known whether this is due to a switch from coupled translation and replication to assembly, or due to overall effects on cellular translation machinery due to the initiation of death programs in infected cells."

7. Figure 6C. There is a difference at 0 units of IFN-[illegible]2 to begin with, is the data normalized to this difference/how significant is the data for increased concentration?
   We appreciate the reviewers' point that this is not entirely clear right now. We have now included the data normalized to the untreated control, and relocated the non-normalized data to the supplemental figure.

<u>Minor comments</u>

1. Line 39, revise to "The roles of viral proteins in these processes have been studied……"
   We have changed this.
2. Line 243, define "NLS, M9-NLS, RS-NLS, and NES".
   We agree that this lacked context and have changed it to read:
   "Nuclear import mediated by several different types of nuclear localization signals (NLSs), as well as the classical nuclear export pathway are affected[6], leading to…"
3. Line 267, correct the typo of "CBV3 and CBV3-2Awt" to "CVB3 and CVB3-2Awt"
   We have changed this.
4. In Figure 6B, the p-IRF3 blots appear to be significantly cropped. Could the authors provide the full, un-cropped images for transparency and clarity?
   We see the reviewer's point and have now reduced the crop to make it better visible. The full uncropped blots are in the raw data.
5. Figure 5. Why was 16hpi omitted for 3CDcs2Awt?
   This is because upon infection at high MOI with 3CDcs-2Awt, very few cells are left alive at 16 hpi.
6. Line 390, correct the typo of "indictaed".
   We have changed this.
7. Line 451 and 252. Omit "both".
   We have changed this.
8. Line 508-509, Figure 7E does not show phase 5.
   We have removed this reference to Fig. 7E.

Reviewer 2 (anonymous)
<u>Points from Summary</u>

In this manuscript van Kuppeveld and colleagues have queried the essential roles of enterovirus 2A proteinase in the initial establishment of infection. Analogous to prior work with mengovirus, the authors created novel variant CVB3 viruses to test downstream roles of 2Apro after the most essential role of 2Apro, to cleave the capsid domain away from nonstructural proteins co-translationally, was mimiced by three approaches. These were inserting an peptide sequence with an 3Cpro cleavage site, inserting a self cleaving peptide from and insect virus, or a second IRES from Theilers virus, all to to separate capsid from nonstructural proteins. In addition, three point mutations were inserted to catalytically inactivate 2Aproteinase. The mutant viruses without 2Apro were then compared in a series of experiments versus identical virus with active 2Apro or WT virus to assess the role of the protease in the virus life cycle.

The three constructs produced a virus that replicates but was modestly defective in single step growth curves in HeLa cells, achieving slightly lower virus replication with similar kinetics. Infections on other cell lines revealed greater apparent replication defects, the three variants were similar to each other. The authors then completed all the remaining work with just one CVB3 variant containing the extra 3Cpro cleavage site.

Through several figures the author test and find large replication and pathogenesis defects in virulence in an SJL mouse model for pancreatitis. The authors then turn back to HeLa cells for remaining analysis.

The next series of mutant 2Apro/replication defects described can be ascribed to functions previously associated with 2Apro in many publications. Many of the findings have been reported previously using other approaches, including blockage of nucleocytoplasmic transport, translation shutoff, hampering stress granule formation, apoptosis and the IFN response. The authors include an interesting experiment showing that IFN paracrine signaling can be overcome by 2Apro. Much of this work is confirmatory, but not new, however confirmations by a new approach is valuable.

The authors use a single-molecule live imaging system to demonstrate the importance of 2Apro in the initial round of replication. In many ways the most interesting part of the manuscript is in Figs 7 and 8 where the single cell analysis system VIRIM is utilized. This is a two-component system consisting of cells expressing a single-chain variable antibody fragment fused to a fluorescent protein (SunTag-antibody, STAb-FP) and viruses that have been genetically engineered to express a short suntag peptide array at the N-terminus of the viral polyprotein. The authors used this approach to separate the initial translation phase from replication and secondary translation/replication phases of infection in single cells and this approach can be powerful.
In these experiments the authors show that 2Apro facilitates the primary round of virus replication, though no direct evidence for rescue of initial (phase 1) virus translation by 2Apro was shown. The authors showed by following intensity of VIRIM translation suntag spots, that 2Apro progressively stimulates viral translation efficiency as the infection proceeds.

The authors do point out the limitations of analysis of 2Apro vs 3Cpro functions in the discussion in regards to regulation of stress granule (formation and resolution), where roles for both proteins have been reported.

Overall the manuscript is well written, methodically laid out and complete. The approach using 2Apro-deficient viruses show that 2Apro is critical for relieving replication bottlenecks in the initial round of replication of infecting virus RNA, subsequently blocking antiviral responses and promoting favorable intracellular conditions for replication. Multiple mechanistic aspects of the replication cycle and virus-host interactions were interogated. The use of a novel virus construct approach largely corroborates many prior findings in the field, but adds some new twists. The single cell replication approach here is novel and could be powerful.

<u>Comments</u>
Conclusions of roles of 2A vs 3Cpro in SG formation is a bit strong. The 2Apro defective mutants have seriously delayed kinetics of 3Cpro cleavage of G3BP compared to WT virus. This could be a clue to a role for other 2Apro functions providing a modified G3BP1 substrate for 3Cpro cleavage, ie after ribosome runoff, loss of interactions with eIF4G or other proteins, etc. Interestingly, a similar delay in kinetics of 3Cpro cleavage of MAVS was also seen with the 2Amut virus and could be discussed.

We acknowledge the reviewer's point that the conclusions on the relative importance of 2Apro and 3Cpro in countering SG formation were worded quite strongly, and we have toned these down. Also, we have added a sentence to discuss the possibility that initial 2Apro cleavage might be important for later 3Cpro-mediated effects on G3BP1 and PKR cleavage:
"… , albeit with considerably delayed kinetics, …"

“However, we cannot exclude the possibility that the delayed kinetics of PKR and G3BP1 cleavage in CVB3-2Amut infection might shield a putative role of 3C^{pro} in countering SGs during CVB3-2Awt infection.”

Also we added the following sentence to the paragraph about MAVS cleavage:
“This implies that either 3C^{pro}-mediated cleavage of MAVS is relatively inefficient and only occurs very late in infection, or that initial 2A^{pro}-mediated cleavage is required to facilitate further proteolytic cleavage by 3C^{pro}.”

In the discussion we have included the following sentence:
“This could also imply a potential novel role for 2A^{pro}, in facilitating efficient cleavage of G3BP1 via an unknown mechanism. Alternatively, … “

Reviewer 3 (anonymous)

Points from Summary

This manuscript describes in detail the numerous aspects of enterovirus 2A protease in the infectious cycle. A variety of experimental and novel technological approaches are being used to study the function of 2A in viral genomes that carried an IRES instead of active 2A or T2A cleavage sites. It was noted that 2A is important for virus virulence of coxsackievirus B3 in a mouse model, is essential for disrupting nucleocytoplasmic transport, stress granule formation and overcoming interferon-induced restriction factors. Finally, it was discovered that 2A is essential during the initial rounds of replication.

Comments

1. Show the abundances of 2A by Western blot analyses.
   We have performed this experiment and included this data in Fig. S2.

2. Are cleavages of cellular transcription factors part of 2A’s function?
   We have not investigated cleavage of cellular transcription factors in this manuscript, but a number of transcription factors were suggested to be cleaved by 2Apro recently (Saeed et al., 2020, PLOS Pathogens). Notably, 3Cpro has been reported to cleave several cellular transcription factors (TATA-binding protein (TBP), CREB, TFIIIC (Das et al., 1993, J. Virol; Clark et al., 1993 Mol. Cell. Biol.; Yalamanchili et al., 1997, J. Virol). Moreover, it should be noted that the NCTD, which is thought to counter mRNA export from the nucleus, and the host translation shutdown, efficiently abrogate expression of cellular genes.

3. Discuss potential dominant-negative effects of 2A on the observed phentypes.
   If we understand this point correctly, the reviewer would like us to discuss any putative effects that the mutant 2Apro regardless of its catalytic inactivity might still have on processes in the cell. To this end, we have added the following sentence to the limitations section:
   “Even though 2A^{pro} is catalytically inactive during CVB3-2Amut infection, the protein is still made, and thus we cannot exclude that it might exert some dominant-negative effects on both viral- and host processes. For example, previous studies successfully used catalytically inactive 2A^{pro} for pull-down studies (Saeed et al., 2020, PLOS Pathogens), demonstrating its continued ability to bind substrates, potentially affecting their function.

4. Adding a Table that lists the functions of proteinases 3C and 2A would be helpful.
   We have now added an overview figure to our discussion (Fig. S8) that represents the different functions of 2Apro and 3Cpro.
